# Artificial Intelligence for Predicting Lung Immune Responses to Viral Infections: From Mechanistic Insights to Clinical Applications

**DOI:** 10.3390/v17111482

**Published:** 2025-11-07

**Authors:** Claudio Tana, Massimo Soloperto, Giampiero Giuliano, Giorgio Erroi, Antonio Di Maggio, Cosimo Tortorella, Livia Moffa

**Affiliations:** 1 Internal Medicine Unit, Eastern Hospital, ASL Taranto, 74024 Manduria, Italy; 2 Internal Medicine Unit, University Hospital of Taranto, 74123 Taranto, Italy; 3 Infectious Disease Unit, University Hospital of Chieti, 66100 Chieti, Italy

**Keywords:** artificial intelligence, viral infections, lung immunity, machine learning, respiratory medicine, SARS-CoV-2, influenza, predictive models, precision medicine

## Abstract

Artificial intelligence (AI) is increasingly transforming biomedical research and patient care by integrating complex biological, radiological, and healthcare information. In the field of viral respiratory infections, AI-driven approaches have shown great promise in elucidating the complexity of lung immune responses and the dynamic interplay between host and pathogen. Applications include predicting cytokine storm and acute respiratory distress syndrome (ARDS), integrating imaging findings with immunological and laboratory data, and identifying molecular and cellular signatures through single-cell and multi-omics analyses. Similar methodologies have been applied to influenza and respiratory syncytial virus (RSV), providing insights into the mechanisms distinguishing protective from maladaptive pulmonary immunity. This narrative review summarizes current evidence on how AI can evolve into a form of translational intelligence, capable of bridging mechanistic immunology with clinical application. The review explores AI-based models for disease severity prediction, patient stratification, and therapeutic response assessment, as well as emerging approaches in drug repurposing and vaccine response prediction. By integrating biological complexity with clinical context, AI offers new opportunities to uncover immune signatures predictive of antiviral or immunomodulatory efficacy and to guide personalized management strategies.

## 1. Introduction

### 1.1. Burden of Viral Respiratory Infections

Respiratory viral infections such as influenza, respiratory syncytial virus (RSV), and coronaviruses including SARS-CoV-2 represent one of the major global health burdens, responsible for millions of hospitalizations and deaths each year across all age groups [1,2,3,4,5]. In Europe, seasonal influenza accounts for approximately 16,000–39,000 respiratory deaths annually, mostly in older adults [1], while in the United States, it causes 9–35 million illnesses and up to 20,000 deaths each year [2]. The continuous gradual mutations in surface glycoproteins (antigenic drift) and major genetic reassortment creating novel viral subtypes (antigenic shift) undermine long-term vaccine effectiveness and complicate both seasonal control strategies and pandemic preparedness [3].

The COVID-19 pandemic caused by SARS-CoV-2 has further underscored the vulnerabilities associated with emerging respiratory viruses, revealing critical gaps in our ability to predict disease trajectories and clinical outcomes at the individual patient level. Beyond its immediate healthcare and societal consequences, the pandemic emphasized how the interplay between viral factors, host immune responses, and comorbidities determines disease severity and long-term sequelae. It also highlighted the need for integrative, data-driven approaches such as AI to identify early predictors of deterioration, optimize resource allocation, and improve precision in therapeutic strategies [6].

At the same time, other respiratory pathogens continue to contribute substantially to the global burden of disease. Despite increasing recognition of the burden of RSV in adults, surveillance and diagnostic strategies are still heterogeneous across regions, leading to a likely underestimation of its true clinical impact [7].

While viral replication initiates infection, the severity of respiratory viral diseases is primarily driven by the host immune response, in which excessive inflammation, endothelial injury, and microvascular thrombosis contribute to tissue damage and mortality [8]. Clinical outcomes range from mild illness to acute respiratory distress syndrome (ARDS) and multi-organ failure, with post-viral fibrosis and long COVID further adding to the long-term disease burden [9]. Despite extensive research, reliable predictive tools to identify patients at risk of deterioration remain limited, emphasizing the need for integrative computational approaches that can link immunological mechanisms to clinical outcomes [10].

### 1.2. Rationale and Aim of the Narrative Review

Given the intricate nature of immune dynamics shaped by viral evolution, coinfections, and interindividual variability, artificial intelligence (AI) provides a powerful framework to analyze high-dimensional, multimodal data and uncover hidden relationships between molecular mechanisms and clinical outcomes, thereby advancing precision medicine in viral immunopathology. This review aims to examine how AI-driven approaches can integrate immunological, molecular, and clinical information to elucidate and predict lung immune responses to viral infections. In this context, we introduce the concept of translational intelligence, defined as the ability of AI to integrate mechanistic, molecular, and clinical knowledge into a unified analytical framework capable of transforming complex data into clinically actionable insights. This approach aims to bridge experimental immunology and patient-centered medicine, fostering a continuum between biological discovery and therapeutic application.

**Figure 1:**
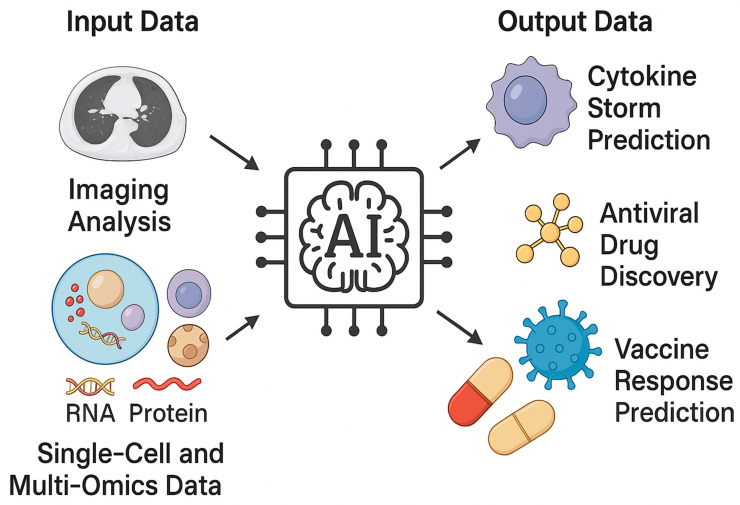
The figure illustrates the conceptual workflow of AI applications in viral respiratory research. Input data including imaging analysis and single-cell or multi-omics datasets feed into the central AI analytical core, which integrates and interprets complex biological information. From these inputs, AI generates output predictions such as cytokine storm assessment, disease severity stratification, antiviral drug discovery, and vaccine response prediction. Together, these applications highlight the translational potential of AI to bridge molecular immunology with clinical decision-making in viral lung infections.

A conceptual overview of how artificial intelligence integrates multi-level biological and clinical data to enable translational applications in viral respiratory infections is depicted in Figure 1.

## 2. Mechanistic Basis of Lung Immune Responses to Viral Infections

### 2.1. Innate Immunity

The respiratory tract represents a frontline interface between the host and the external environment, continuously exposed to airborne microorganisms and particulate matter. Innate immunity constitutes the first barrier against viral invasion, primarily mediated by alveolar macrophages, dendritic cells (DCs), and neutrophils, supported by an intricate network of epithelial and endothelial signaling [11]. Alveolar macrophages act as resident sentinels, rapidly recognizing viral particles via pattern recognition receptors (PRRs) such as Toll-like receptors (TLR3, TLR7, TLR9) and RIG-I–like receptors, leading to the activation of interferon (IFN) pathways and the release of antiviral mediators, including IFN-α/β, IL-6, TNF-α, and CXCL10 [12]. Dendritic cells, especially plasmacytoid DCs, serve as the key bridge to adaptive immunity through antigen processing and migration to draining lymph nodes, where they prime naïve T cells [13].

Neutrophils, recruited through chemokines such as CXCL8 and CCL2, contribute to viral clearance via phagocytosis and neutrophil extracellular traps (NETs), yet their excessive activation promotes oxidative stress and alveolar-capillary barrier injury. Interferon signaling represents a central regulatory node: while timely IFN production restricts viral replication, delayed or excessive responses contribute to immune-mediated lung injury, as observed in severe influenza and COVID-19 [14].

Age-related immune remodeling further complicates these dynamics. In elderly people, immunosenescence leads to attenuated PRR signaling, reduced IFN-α/β secretion, and impaired antigen presentation due to decreased MHC-II and co-stimulatory molecules [11]. These alterations delay pathogen recognition and favor viral persistence, setting the stage for secondary bacterial infections.

### 2.2. Adaptive Responses

Following innate activation, adaptive immunity orchestrates pathogen-specific clearance.

CD8^+^ cytotoxic T lymphocytes (CTLs) eliminate infected epithelial cells through perforin- and granzyme-dependent mechanisms, while CD4^+^ helper T cells regulate the magnitude and quality of these responses and support B-cell activation in secondary lymphoid organs [15]. However, with advancing age and chronic inflammation, thymic involution limits the generation of naïve T cells, narrowing T-cell receptor (TCR) diversity and promoting the accumulation of exhausted, terminally differentiated clones [16]. The imbalance between Th1, Th2, and Th17 subsets alters cytokine polarization favoring IL-4 and IL-17 at the expense of IFN-γ. and impairs effective antiviral control [17].

In parallel, B-cell responses are blunted through defective germinal center formation, reduced class-switch recombination, and suboptimal somatic hypermutation, resulting in antibodies of lower affinity and reduced mucosal IgA secretion [18]. The accumulation of so-called age-associated B cells (ABCs) expressing T-bet and CD11c adds further dysregulation, as these subsets secrete proinflammatory cytokines while providing limited protective immunity [19]. Together, these phenomena converge toward a phenotype of adaptive immune exhaustion, delaying viral clearance and heightening the risk of prolonged or recurrent infection.

### 2.3. Dysregulated and Pathological Responses

When innate and adaptive responses become excessive or uncoordinated, the lung microenvironment shifts from defense to immunopathology. Hyperactivation of NF-κB and the NLRP3 inflammasome drives uncontrolled cytokine production, especially IL-1β, IL-6, and TNF-α, leading to endothelial dysfunction, alveolar edema, and ARDS [20]. Persistent inflammation promotes infiltration of monocyte-derived macrophages and neutrophils, excessive ROS release, and mitochondrial dysfunction, perpetuating tissue injury and fibrosis [21].

The accumulation of senescent pulmonary epithelial and endothelial cells, which acquire a senescence-associated secretory phenotype (SASP), further amplifies local inflammation through IL-8, TGF-β, and metalloproteinases [22]. These mediators contribute to extracellular matrix remodeling and post-viral fibrotic sequelae, a process described in both SARS-CoV-2 and influenza survivors [23]. Moreover, persistent inflammasome activation and inadequate resolution signaling (e.g., defective IL-10 and TGF-β regulation) hinder tissue repair, prolonging recovery and fostering chronic pulmonary dysfunction [24].

### 2.4. Knowledge Gaps and Predictive Modeling

Despite major advances, several knowledge gaps remain. The molecular determinants of age- and comorbidity-related susceptibility to viral pneumonia are incompletely understood, particularly the integration between immunosenescence, metabolic rewiring, and lung microenvironmental cues [11]. Moreover, interindividual variability in interferon responses and epigenetic modulation of antiviral genes (e.g., via DNA methylation drift and miRNA dysregulation) represent promising but underexplored predictive biomarkers [25].

Emerging predictive modeling and systems immunology approaches aimed at integrating omics data, immune phenotyping, and clinical variables may enable early identification of patients at risk of hyperinflammatory or fibrotic trajectories. Machine learning (ML) frameworks applied to transcriptomic and proteomic datasets could refine personalized therapeutic strategies, from interferon modulators to senolytics and immunonutrition, aimed at restoring immune equilibrium and preserving lung architecture in vulnerable populations [26].

## 3. AI and Computational Approaches for Modeling Immune Responses

### 3.1. Machine Learning (ML) Methods: Regression, Random Forest, SVM

The main ML approaches applied in this field include ensemble algorithms such as Random Forest and XGBoost, which are valued for robustness and interpretability, and Deep Learning (DL) architectures capable of identifying complex non-linear patterns across omics and imaging data [26]. Graph neural networks (GNNs) are increasingly used to model molecular and cell–cell interaction networks. Model performance is typically assessed using metrics such as AUC/AUROC, AUPR, F1 score, and Matthews correlation coefficient (MCC), with higher values (e.g., AUROC > 0.8) generally indicating strong predictive reliability [26]. The F1 score represents the harmonic mean of precision and recall, while the MCC provides a more balanced assessment of model performance, particularly in imbalanced datasets [26].

Classical ML methods have become essential tools for decoding complex immunological and clinical datasets and for predicting disease states or outcomes. These models leverage quantitative features such as immune receptor sequences (BCR, TCR), serum biomarkers, immune cell counts, and imaging-derived variables to identify multivariate signatures of immune activation and disease progression [26].

A recent landmark example is the Mal-ID (Machine Learning for Immunological Diagnosis) framework, which applied ML techniques to BCR/TCR repertoire data from 593 individuals encompassing a broad spectrum of immune conditions, including COVID-19, HIV, systemic lupus erythematosus, type 1 diabetes, vaccination responses, and healthy controls [27]. Using gradient-boosted decision trees and neural embedding models trained on millions of receptor sequences, Mal-ID achieved mean multiclass AUROC values of 0.89–0.96 across 11 diagnostic categories. When evaluated in independent external validation cohorts, model performance remained robust (average area under the receiver operating characteristic curve, AUROC ≈ 0.90), demonstrating strong generalizability beyond the training dataset. Feature attribution analyses revealed that antigen-specific CDR3 motifs and V/J gene usage patterns were the most informative features for classification, with distinct sequence signatures emerging for autoimmune versus infectious conditions [27]. Notably, in COVID-19, Mal-ID identified shared clonotypes enriched for IGHV3-53 germline genes, consistent with experimentally characterized SARS-CoV-2-neutralizing antibodies. Importantly, the study showed that receptor repertoires alone (without clinical or demographic covariates) carry sufficient immunological information to enable diagnosis with high accuracy. These findings illustrate how ML could translate large-scale adaptive immune receptor data into clinically interpretable, generalizable diagnostic tools [27].

Another example is the multi-modal ML framework for COVID-19 detection developed by Tur et al., which combined clinical biomarkers (e.g., C-reactive protein, ferritin, D-dimer) with chest X-ray imaging features to distinguish SARS-CoV-2–positive from negative cases. The study evaluated multiple architectures and demonstrated that the Gradient Boosting + VGG fusion model achieved the best overall performance, with an AUROC of 0.94, F1-score of 0.93, specificity of 93%, negative predictive value (NPV) of 96%, and a MCC of 0.91. Interpretability analysis using SHAP (SHapley Additive exPlanations) and LIME (Local Interpretable Model-Agnostic Explanations) confirmed that CRP, ferritin, and radiomic lung patterns were among the most influential predictors, underscoring the biological plausibility of the model’s decisions. It should be noted that the goal of these approaches is not to replace molecular diagnostic tests, which remain the gold standard for pathogen detection, but rather to complement them by enabling rapid triage, integration of imaging and clinical data, and early risk assessment in settings with limited diagnostic resources or high patient volume.

These findings further illustrate how multimodal fusion frameworks could effectively integrate heterogeneous immunological and imaging information to enhance diagnostic precision and provide biologically interpretable insights in viral respiratory infections [28].

Similarly, the BIO-CXRNET study introduced a multimodal stacking approach combining chest X-ray (CXR) features and clinical biomarkers to predict mortality risk among COVID-19 patients. Using a dataset of 930 patients, the multimodal model achieved precision of 89.03%, sensitivity of 90.44%, and F1-score of 89.03% in risk stratification (low vs. high risk). This fusion strategy improved accuracy by about 6% relative to using either modality alone. For outcome prediction (survival vs. death) among high-risk patients, the model delivered accuracy of 92.3%, surpassing individual models based on CXR images (89.5%) or clinical data (90.11%) alone. The AUC reported for development and validation cohorts were 0.981 and 0.939, respectively. Feature selection via random forest identified LDH, O_2_ saturation, WBC count, age, and CRP as key predictors, and a nomogram combining these with the CXR score yielded an F1 of 92.88% for high-risk death prediction [29].

Together, these studies illustrate that even relatively classical ML frameworks such as ensemble classifiers, penalized regression, and feature-based support vector machines remain powerful and interpretable tools for modeling immune responses. They provide reproducible, data-driven insight into host–pathogen interactions and enable clinically actionable predictions from multi-dimensional immune and clinical datasets.

### 3.2. Deep Learning (DL): Neural Networks for Complex Non-Linear Relationships

DL excels at capturing hierarchical, non-linear patterns in high-dimensional immune datasets (e.g., single-cell omics, receptor sequences, imaging). For example, Maslova et al. developed a convolutional neural network (CNN) architecture, AI-TAC, trained on regulatory DNA sequences associated with chromatin accessibility across 81 immune cell types. The model learned to predict cell-type-specific openness of chromatin solely from DNA sequence, rediscovered known transcription factor motifs (e.g., Pax5, Ebf1), and revealed combinatorial regulatory logic underlying lineage decisions. The network’s feature attribution recapitulated established regulatory hierarchies (e.g., Pax5/Ebf1 in B cells, Spi1/Cebp in myeloid) and stage-specific motif interactions (such as Pax5/Prdm1) [30].

DL models that integrate sequence embeddings and structural encodings have shown potential for predicting adaptive immune receptor specificity from BCR and TCR sequence data. These architectures aim to capture the complex sequence–structure relationships that underlie antigen recognition. Although still limited by data availability and annotation depth, such approaches have demonstrated improved performance over conventional motif-based or alignment-based classifiers in identifying receptor–antigen binding patterns. This highlights the emerging promise of embedding-based representations for advancing immunological inference and repertoire analysis [31].

### 3.3. Systems Biology and Network-Based AI: Modeling Host–Virus Interaction Networks

Systems approaches integrate multi-omic and protein interaction data to reconstruct virus–host interactomes and identify critical pathways perturbed during infection. These network models allow quantitative inference of key nodes, hubs, and druggable targets, supporting both mechanistic understanding and therapeutic repurposing [26].

PHISTO (Pathogen–Host Interaction Search Tool) is a comprehensive, manually curated database designed to catalog experimentally validated protein–protein interactions (PPIs) between human hosts and a wide range of pathogens. The resource integrates data from major repositories including IntAct, DIP, MINT, BioGRID, and HPIDB, and as of its initial release contained over 20,000 interactions involving 300 pathogenic organisms. Each entry is annotated with UniProt identifiers, experimental methods, literature references, and network topology metrics such as degree and betweenness centrality, enabling systematic exploration of host–pathogen interactomes. PHISTO supports both retrieval and visualization of PPI networks, providing a foundation for network-based inference, machine learning, and drug repurposing analyses. It has since become a benchmark dataset for graph-based prediction of virus–host interactions and identification of host dependency factors across multiple viral systems [32].

Madan et al. introduced STEP (Siamese Tailored deep-sequence Embedding of Proteins), a transformer-based DL model for predicting virus–host protein–protein interactions (PPIs) using only primary amino acid sequences. The method integrates ProtBERT embeddings, which is trained on over 2 billion protein sequences, with a Siamese neural-network architecture and a positive–unlabeled (PU) learning scheme [33].

Across benchmark datasets (human–virus, yeast, and human PPIs), STEP achieved near–state-of-the-art performance, with AUC values up to ≈0.99 and F1-scores > 0.97, outperforming classical machine learning and LSTM-based models. Applied to biologically relevant cases, STEP identified novel predicted interactions between the JCV VP1 capsid protein and human brain receptors (AUC ≈ 0.89; enrichment in serotonin and tyrosine-kinase signaling pathways) and between the SARS-CoV-2 spike protein and the sigma-2 receptor (TMEM97) (AUC ≈ 0.83), consistent with experimental reports of sigma-receptor-modulating antiviral effects [33].

Explainable-AI analysis (integrated gradients) localized sequence regions driving predictions, improving the interpretability of deep sequence embeddings. The study demonstrates that transformer-based embeddings and Siamese architectures can capture latent biophysical constraints in protein sequences, supporting large-scale, data-efficient prediction of virus–host interactomes and hypothesis generation for antiviral drug discovery [33].

In summary, network-based and AI-driven analyses reveal how viral infections perturb host molecular systems and highlight druggable nodes within these networks. These frameworks lay the groundwork for the next step in integrating multimodal data from omics, imaging, and clinical sources to achieve a more comprehensive understanding of host–virus interactions and therapeutic responses.

### 3.4. Multimodal Integration: Omics (Transcriptomics, Proteomics, Metabolomics), Imaging (HRCT, Radiomics), and Clinical Data

The integration of molecular, imaging, and clinical data is increasingly used in computational studies of host–virus interactions and disease progression. Single-modality analyses often fail to capture the complexity of immune dysregulation, whereas combining multi-omics and radiomics data offers complementary information on cellular pathways and tissue-level changes. Recent developments in matrix factorization, manifold alignment, and deep generative models have enabled more coherent representations of heterogeneous datasets, supporting cell-state identification, trajectory inference, and biomarker discovery in large-scale immune analyses. These integrative frameworks also improve data harmonization and interpretability, particularly when combined with attention-based and explainability methods such as Shapley-value attribution [34].

Among applied studies, Shiri et al. developed a four-class CT-based severity model for COVID-19 using data from 1110 patients stratified by radiological and clinical criteria. Entire lung volumes were segmented to extract quantitative radiomic features, followed by feature selection via bagging random forest (BRF) and multivariate adaptive regression splines (MARS) coupled with multinomial logistic regression classifiers. The BRF + MLR model achieved the best performance, with precision = 0.86, recall = 0.85, accuracy = 0.92, and AUC = 0.85 (95% CI 0.81–0.89) on the held-out test set, significantly outperforming MARS + MLR (AUC = 0.81). Key predictive descriptors included first-order texture metrics (mean absolute deviation, skewness, variance) and gray-level co-occurrence matrix features related to tissue heterogeneity. These findings demonstrate that radiomic signatures extracted from routine CT imaging, when coupled with robust machine learning pipelines, can accurately stratify COVID-19 pneumonia severity, supporting their use as non-invasive markers for clinical risk assessment and outcome prediction [35].

Complementing these severity models, Chen et al. developed a support vector machine (SVM)-based CT radiomics model to distinguish COVID-19 from non-COVID pneumonia using 326 chest CT scans from 134 patients. Radiomic, radiological, quantitative, and clinical features were extracted and combined into an integrated classifier. The multimodal SVM model achieved the highest levels of accuracy, sensitivity and specificity on the independent testing cohort, clearly outperforming single-feature models based on radiomic or clinical variables alone. These results underscore the additive value of combining imaging-derived radiomic signatures with traditional radiological and clinical parameters, enhancing diagnostic precision and reducing the risk of misclassification in early COVID-19 diagnosis [36].

In parallel, Hardy-Werbin et al. developed MultiCOVID, a multimodal DL framework that integrates CXR imaging and blood test data to discriminate between COVID-19, heart failure, non-COVID pneumonia, and healthy controls. The study analyzed 8578 samples from 6123 patients (mean age 66 ± 18 years) and trained open-source neural network architectures on paired imaging–laboratory data. The resulting multimodal model achieved an overall accuracy of 84% and a mean AUC of 0.92 (95% CI 0.89–0.94). In a subset of 300 randomly selected cases, MultiCOVID significantly outperformed five expert thoracic radiologists, with an accuracy of 69.6% vs. 43.7–58.7% (*p* < 0.001). These findings highlight the synergistic diagnostic value of combining imaging and hematological biomarkers within a unified AI framework and underscore the translational potential of multimodal DL for clinical triage and decision support [37].

Collectively, evidence from radiomics, omics, and integrative ML confirms that multimodal data fusion markedly enhances diagnostic accuracy, prognostic power, and mechanistic interpretability in viral and post-viral disease. By coupling molecular and cellular signatures with quantitative imaging and clinical metadata, these approaches can capture the full continuum of host–virus interactions from immune activation to tissue remodeling thereby supporting precision diagnostics, risk stratification, and personalized follow-up.

### 3.5. Explainable AI (XAI): Importance for Biological Interpretation and Clinical Trust

As predictive models in infectious and inflammatory diseases grow more complex, the need for explainable artificial intelligence (XAI) has become critical to ensure transparency, clinical trust, and regulatory acceptance. XAI methods aim to clarify how models such as DL and multimodal architectures derive their predictions from heterogeneous data sources such as omics, imaging, and clinical variables [38].

A recent systematic review and meta-analysis of 62 studies (2018–2025) examined the application of XAI techniques within clinical decision support systems (CDSSs) across diverse domains, including radiology, oncology, neurology, cardiology, and intensive care. The most frequently adopted approaches were SHAP (33%), Grad-CAM (27%) and LIME (20%), while emerging frameworks incorporated attention-based and counterfactual explanations. Overall model performance was strong (median AUC = 0.87, accuracy = 86%), and the inclusion of quantitative interpretability metrics was associated with an increase in clinician trust. However, only 29% of studies had complete databases, and fewer than one-third ensured code transparency or reproducibility. Common limitations included the lack of standardized fidelity metrics, limited bias and fairness assessment, and insufficient integration with electronic health records. The authors conclude that although XAI adoption in healthcare is expanding rapidly, clinical translation remains fragmented; integrating robust attribution methods (e.g., SHAP, Grad-CAM) with clinician-centered validation and standardized benchmarking will be crucial to achieving transparency, reliability, and regulatory readiness for multimodal AI systems in clinical practice [39].

### 3.6. Critical Appraisal of Current AI Evidence and Biological Novelty

Although numerous AI models have been proposed for the analysis of immune and clinical datasets, relatively few have undergone rigorous external or prospective validation. Among the most robust frameworks, ensemble learning algorithms such as XGBoost and Random Forest have consistently demonstrated high reproducibility and generalizability across independent cohorts, particularly in predicting ARDS, sepsis outcomes, and multimodal risk stratification [40]. By contrast, DL architectures, while powerful in capturing non-linear relationships often remain limited by small sample sizes, lack of standardization, and scarce clinical benchmarking [33].

From a biological perspective, AI-driven analyses have yielded genuinely novel insights rather than merely confirmatory findings. Examples include the identification of IGHV3-53 and IGHV1-69 BCR clonotypes associated with neutralizing responses in COVID-19, the discovery of sigma-2 receptor (TMEM97) interactions with SARS-CoV-2 spike protein through transformer-based sequence embeddings, and the delineation of chromatin-accessibility motifs driving immune-cell differentiation (e.g., Pax5/Ebf1 and Spi1/Cebp) [27,30,33]. These advances demonstrate that, when properly validated, AI can transcend correlation-based modeling to generate mechanistically informative hypotheses that complement experimental immunology.

Nevertheless, translation to clinical practice requires harmonized datasets, transparent pipelines, and standardized evaluation metrics to ensure that algorithmic predictions correspond to biologically and clinically meaningful outcomes.

## 4. AI for Predicting Disease Severity and Outcomes

### 4.1. Predicting Cytokine Storm and ARDS Risk

AI models can anticipate hyper-inflammatory deterioration and impending ARDS hours to days in advance by integrating routine clinical data, inflammatory biomarkers (IL-6, CRP, ferritin, D-dimer, lymphocyte count), and imaging features. Lin et al. developed an ML model to predict both the occurrence and mortality of non-pulmonary sepsis–associated ARDS using data from the MIMIC-IV database, which includes detailed clinical information from more than 65,000 intensive care unit (ICU) patients. The cohort included 11,409 sepsis patients without pre-existing pulmonary disease, of whom 66.9% developed ARDS. The investigators compared several algorithms including K-nearest neighbor, support vector machine, decision tree, deep neural network, and XGBoost, and found that XGBoost achieved the best performance. In internal validation, the model predicted ARDS onset and 28-day mortality with accuracies of 77.5% and 71.8%, respectively; in external validation, these accuracies rose to 78.0% and 81.4%. Patients who developed ARDS had longer ICU stays (6.2 ± 5.2 vs. 4.4 ± 3.7 days, *p* < 0.01) and higher mortality (19.5% vs. 14.9%, *p* < 0.01). This study demonstrates that ML can reliably identify high-risk sepsis patients before ARDS develops, providing a framework for early warning and individualized management [40].

CXR and CT-based DL models can add value by quantifying the extent and texture of alveolar damage in ARDS. In a multicenter study, a CNN trained on more than 595,000 chest radiographs achieved expert-level performance for ARDS detection, with an AUROC of 0.92 (95% CI 0.89–0.94) in the internal test set and 0.88 (95% CI 0.85–0.91) in the external validation cohort. When applied to cases reviewed by six independent physicians, the model reached 83% sensitivity and 88% specificity, demonstrating a comparable accuracy to specialist interpretation [41]. These findings indicate that AI-driven imaging analysis can objectively identify radiographic patterns of alveolar damage and, when fused with EHR-derived clinical and biomarker data, multimodal systems could achieve superior discrimination for near-term ARDS (6–24 h) and hyper-inflammatory (cytokine storm) phenotypes [42].

Operationally, top predictive signals include rising oxygen requirements (SpO_2_/FiO_2_ decline), tachypnea and increased work of breathing, escalating CRP and IL-6 with lymphopenia and elevated D-dimer, positive fluid balance, and early renal or hemodynamic dysfunction. These clinical and laboratory markers mirror endothelial activation, cytokine overproduction, and microvascular injury, processes central to the pathogenesis of ARDS [43]. In a recent large-scale sepsis–ARDS machine learning model, Zhou et al. analyzed over 10,000 patients from the MIMIC-IV database and demonstrated that circulating immune and biochemical parameters were strong determinants of ARDS development. The XGBoost algorithm achieved the best predictive performance (AUC = 0.764), with monocyte and neutrophil counts, mean arterial pressure, pH, and platelet count emerging as the top-ranked features, each reflecting inflammatory load, metabolic stress, and microcirculatory compromise. Using SHAP, the authors further showed how variations in these variables modulated ARDS risk, underscoring the prognostic utility of dynamic biomarker monitoring and its potential integration into early warning AI systems for critically ill septic patients [44].

### 4.2. Identifying Patients at Risk of Severe Influenza Pneumonia or RSV Bronchiolitis

ML approaches have recently shown strong potential in predicting clinical deterioration and severe outcomes among patients with viral respiratory infections, particularly influenza and RSV. In a large multicenter study, Tan et al. applied ML models to predict adverse outcomes in 5508 older adults (≥65 years) presenting to emergency departments with confirmed influenza. Using ten routinely collected EHR variables and a 70/30 train–test split, the models achieved high discriminative performance with AUCs of 0.840 for hospitalization, 0.765 for pneumonia, 0.857 for sepsis or septic shock, 0.902 for ICU admission, and 0.889 for in-hospital mortality. The random forest and XGBoost algorithms yielded the best predictive results, outperforming traditional logistic regression models. Importantly, the predictive tool was integrated into the hospital information system to assist real-time clinical decision-making, highlighting the practical feasibility of ML deployment in emergency settings. These findings emphasize that even using a limited set of routinely available clinical data, ML can provide accurate and timely risk stratification in older adults, who are particularly vulnerable to complications such as severe pneumonia, sepsis, or acute respiratory failure [45].

Similar approaches could be extended to RSV bronchiolitis and other viral infections, enabling early escalation of care and optimization of resource allocation. A predictive model developed on data from twelve consecutive respiratory seasons evaluated 243 adults aged ≥60 years with laboratory-confirmed RSV infection and medically attended respiratory illness. Among these patients, 19% experienced serious outcomes, including hospital admission, emergency department visits, or pneumonia. Using logistic regression with penalized maximum likelihood estimation, the reduced model identified age ≥75 years, prior emergency department visits, crackles or rales, tachypnea, wheezing, new or increased sputum, and new or worsening dyspnea as significant predictors of disease severity. The model achieved a sensitivity of 66.0% and specificity of 81.6%, showing moderate but clinically useful discrimination for early identification of older adults at high risk of RSV-related complications. These findings suggest that even simple models based on clinical parameters can assist in triage and early intervention, while future AI and ML frameworks combining these features with biomarkers and imaging data could further refine prognostic accuracy and improve patient management [46].

### 4.3. AI Models for Mortality Prediction, ICU Admission, Ventilatory Support Needs

A limited but growing number of studies have applied AI and ML techniques to predict mortality, ICU admission, and ventilatory support needs in critically ill patients. Recent DL models trained on large ICU cohorts have shown high discriminative power for mortality prediction among mechanically ventilated patients, clearly outperforming conventional severity scores. In a large-scale study using data from over 25,000 ICU patients requiring mechanical ventilation, an AI-based model achieved an AUROC of 0.862, with an accuracy of 0.789 and an F1-score of 0.747, representing a substantial improvement over previous benchmarks (AUROC 0.821). The model demonstrated excellent calibration and generalization, highlighting hemodynamic instability, oxygenation indices, renal dysfunction, and inflammatory biomarkers as the most relevant mortality predictors [47], confirming the ability of ML frameworks to anticipate poor outcomes in mechanically ventilated patients and support early clinical decision-making, improving triage and risk stratification in ICU settings [47].

Other studies have focused on early mortality prediction upon ICU admission, particularly in patients with sepsis. In a cohort of adult ICU patients with sepsis, a Random Forest model trained on 38 clinically relevant variables achieved an AUROC of 0.94 (±0.01). Among the most predictive features were the Sequential Organ Failure Assessment (SOFA) score and average urine output, both strong indicators of organ dysfunction and perfusion status. The use of SHAP provided interpretability, revealing that hemodynamic stability, renal output, and systemic inflammation were dominant contributors to outcome risk. These findings highlight the dual advantage of modern ML frameworks such as high predictive accuracy combined with clinical transparency, facilitating bedside applicability and integration into decision-support systems for real-time prognosis and treatment prioritization in sepsis management [48].

AI applications have shown substantial promise also in predicting the need for mechanical ventilation, optimizing ventilator settings, and supporting weaning and extubation decisions among critically ill patients. Across the 37 studies reviewed, DL and ensemble models (particularly Random Forest, XGBoost, and CNN architectures) achieved AUCs ranging from 0.70 to 0.90 for predicting intubation, ventilatory support, and outcomes in respiratory failure [49]. In COVID-19 cohorts, CT and X-ray-based AI tools accurately predicted the need for invasive ventilation (AUC up to 0.87) and mortality when integrated with clinical data [50]. Other systems have demonstrated strong performance in detecting patient–ventilator asynchrony and in estimating key respiratory mechanics, including compliance, CROP index, and maximal inspiratory pressure [51].

In ICU patients undergoing mechanical ventilation, difficult weaning represents a major clinical challenge and a key determinant of adverse outcomes. Failure or delay in discontinuing ventilatory support is associated with an increased risk of ventilator-associated complications, longer ICU and hospital stays, and substantially higher healthcare costs. Early identification of patients at risk is therefore essential to guide timely intervention, optimize sedation and respiratory management, and reduce morbidity [52].

A recent study developed a ML-based risk prediction model to improve the early recognition of difficult weaning. Using data from 487 ventilated ICU patients and 36 routinely collected clinical variables, the Random Forest model achieved a high predictive performance, with an AUC of 0.805, accuracy of 0.748, recall of 0.888, specificity of 0.767, and F1-score of 0.825 [53]. The model successfully identified major determinants of weaning difficulty, integrating physiological, respiratory, and hemodynamic indicators into an interpretable predictive framework. By enabling early risk stratification and individualized treatment planning, this approach can support clinicians in tailoring respiratory management, potentially reducing ventilator dependence, ICU stay, and improving overall patient outcomes.

Collectively, these AI approaches demonstrate how combining temporal EHR data, respiratory indices, and laboratory biomarkers can enhance prognostic precision, allowing clinicians to anticipate deterioration and optimize ICU resource allocation.

## 5. AI for Immune Response Modeling, Vaccine Prediction, and Antiviral Drug Repurposing

### 5.1. Identifying Immune Signatures Predictive of Antiviral or Immunomodulatory Therapy Response

AI is increasingly being applied to accelerate therapeutic target discovery and improve prediction of antiviral and immunomodulatory therapy response in respiratory infections. Systems-level analyses integrating transcriptomic, proteomic, and immunophenotypic data have enabled the identification of immune signatures predictive of antiviral efficacy or cytokine-modulating therapy response. Recent systems vaccinology approaches have demonstrated that early interferon and innate immune gene expression profiles can predict both the magnitude and durability of antibody responses to influenza and SARS-CoV-2 vaccines [54].

### 5.2. Predicting Vaccine Responsiveness and Durability of Immune Protection

Similarly, ML approaches have been successfully applied to integrate multi-omic data, such as transcriptomic, proteomic, and cytokine profiles, to predict vaccine immunogenicity and reactogenicity. By analyzing early post-vaccination gene expression patterns, these models can distinguish individuals likely to develop strong or weak immune responses and anticipate potential adverse reactions. Such systems-level analyses have identified molecular pathways related to interferon signaling, antigen presentation, and metabolic reprogramming as key determinants of vaccine efficacy. These findings could support the use of AI-driven systems vaccinology to optimize vaccine formulation, guide personalized immunization strategies, and improve the prediction of long-term protective immunity [55].

### 5.3. AI-Assisted Drug Repurposing for Viral Pneumonia

AI has also transformed drug repurposing strategies for viral pneumonia, integrating GNNs, knowledge graphs, virtual screening, and network pharmacology frameworks to uncover novel antiviral candidates among approved drugs. Since the onset of the COVID-19 pandemic, several large-scale AI pipelines have been developed to systematically integrate virus–host–drug interactions with in vitro and population-level validation data. In one approach, over 3600 compounds associated with COVID-19 were mapped into a knowledge graph encompassing viral baits, host genes, signaling pathways, and phenotypic effects. A deep GNN model was then trained to infer drug embeddings and prioritize candidates according to their biological connectivity, clinical trial evidence, and real-world outcomes. The resulting pipeline successfully identified 22 high-ranking drugs, including azithromycin, atorvastatin, aspirin, acetaminophen, and albuterol, several of which demonstrated synergistic antiviral or host-modulatory activity in subsequent analyses. These findings highlight how graph-based AI frameworks can accelerate the discovery of repurposable agents for respiratory viral infections by integrating multi-scale biological and clinical evidence [56]. In another approach, a DL framework named AntiViralDL applied a LightGCN to a virus–drug bipartite graph constructed from FDA-approved antiviral associations and the Drugvirus2 database. By leveraging self-supervised contrastive learning and data augmentation in the embedding space of virus and drug nodes, the model effectively addressed data sparsity and improved predictive performance. AntiViralDL achieved an AUC of 0.845 and AUPR of 0.849, outperforming multiple benchmark algorithms and successfully identifying potential anti-COVID-19 drug candidates. This study illustrates how graph-based AI architectures can accelerate antiviral drug discovery and repurposing by integrating heterogeneous molecular and pharmacological information [57]. An overview of the main translational applications of artificial intelligence in viral respiratory infections is presented in Table 1, summarizing the types of data used, methodological objectives, and their current level of clinical implementation.

The table summarizes the he main translational domains where AI has been applied to viral lung infections. Applications are grouped into three categories, Diagnostics, Prognosis, and Drug Discovery, highlighting their data sources, methodological objectives, representative examples, and clinical translational potential. These studies collectively illustrate how AI integrates multimodal data (clinical, molecular, and imaging) to support early diagnosis, risk stratification, and therapeutic innovation in viral respiratory diseases. Abbreviations: AI = Artificial Intelligence; ARDS = Acute Respiratory Distress Syndrome; AUC = Area Under the Curve; AUROC = Area Under the Receiver Operating Characteristic Curve; BCR/TCR = B-cell/T-cell Receptor; CRP = C-reactive Protein; CT = Computed Tomography; DL = Deep Learning; EHR = Electronic Health Record; GNN = Graph Neural Network; ICU = Intensive Care Unit; IL-6 = Interleukin-6; ML = Machine Learning; MV = Mechanical Ventilation; RF = Random Forest; SHAP = SHapley Additive exPlanations; XAI = Explainable Artificial Intelligence; XGBoost = Extreme Gradient Boosting; R&D = Research and Development.

## 6. Conclusions—The Time for Translational Intelligence

Despite the remarkable progress of AI in biomedical research, its application to viral respiratory infections remains limited by methodological, clinical, and ethical challenges [58].

Data fragmentation, small and unbalanced cohorts, and the opacity of DL models continue to restrict generalizability and clinician trust [59]. The rapidly evolving nature of viral infections further complicates the temporal stability of predictions, while unresolved issues around privacy, bias, and accountability hinder real-world integration [11]. Overcoming these barriers requires harmonized data infrastructures, multicenter validation, and explainable, continuously updated models embedded within clinical workflows [60]. Beyond technical refinement, AI must evolve into true translational intelligence, constituting a bridge between immunological mechanisms and clinical practice. The establishment of standardized data formats and open-access repositories will be essential to ensure reproducibility, transparency, and equitable progress in AI-driven biomedical research. In addition, recent regulatory developments such as the EU AI Act and FDA initiatives on AI-based medical tools, underline the necessity of explainable and transparent algorithms to ensure safety, accountability, and clinician trust in real-world healthcare applications [61]. If supported by rigorous prospective trials and interdisciplinary collaboration, AI has the potential to transform the prediction, prevention, and management of viral lung infections, bringing precision medicine closer to everyday care.

## Figures and Tables

**Table 1:** Translational applications of artificial intelligence (AI) in viral respiratory infections, organized by thematic domain. AI Application/Data Sources.

Domain	AI Application/Data Sources	Main Objectives	Key Achievements/Examples	Translational Potential
A. Diagnostics	Prediction of cytokine storm and ARDS Clinical data (vitals, labs), imaging (CT/X-ray), biomarkers (IL-6, CRP, D-dimer) Integration of radiomics and omics Chest CT, proteomic/transcriptomic data Explainable AI (XAI) Multimodal (omics + imaging + clinical)	Early identification of hyperinflammatory deterioration Severity stratification and molecular-imaging correlation Interpretability and clinician trust	ML and DL models predicting ARDS onset with AUC > 0.8; integration of EHR and imaging features improves near-term risk prediction [40,41,42] Multimodal models (BIO-CXRNET, MultiCO- VID) achieve > 90% accuracy for mortality prediction [29,37] SHAP, Grad-CAM, and LIME frameworks enhance transparency and regulatory compliance [38,39]	High—supports real-time triage and early intervention High—enables precision diagnostics and monitoring Essential—prerequisite for adoption in clinical workflows
B. Prognosis/Risk Prediction	Immune repertoire and multi-omics modeling BCR/TCR sequencing, single-cell RNA-seq, proteomics Ventilatory support and weaning prediction ICU physiological data, ventilator parameters AI models for severity, mortality, and ICU admission EHR, biomarkers, respiratory indices	Identification of immune signatures of protective vs. maladaptive responses Predicting need for MV and difficult weaning Early risk stratification and clinical decision support	ML on receptor repertoires (Mal-ID) achieves AUROC ≈ 0.9 for immune condition classification [27] Random Forest AUC ≈ 0.80; DL for ventilator optimization [53] RF and XGBoost models outperform conventional severity scores [45,48]	Moderate–High informs vaccine and therapeutic design Moderate–High—improves ICU resource allocation High—supports real-time prognosis and prioritization
C. Drug Discovery and Therapeutic Optimization	Vaccine response prediction Transcriptomics, proteomics, cytokine profiling AI-assisted drug repurposing for viral pneumonia Knowledge graphs, virus–host–drug networks, clinical data	Forecasting immunogenicity, durability, and reactogenicity Prioritization of repurposable antivirals and immunomodulators	AI models predict antibody magnitude and durability from early innate signatures [54,55] GNN pipelines identify > 20 candidate drugs with validated synergistic effects (e.g., azithromycin, atorvastatin) [56,57]	High—guides personalized immunization and booster strategies High—accelerates therapeutic translation and reduces R&D cost

## Data Availability

No new data were created or analyzed in this study. Data sharing is not applicable to this article.
